# A multi-institutional observational study on the effects of three-dimensional radiotherapy and weekly 40-mg/m^2^ cisplatin on postoperative uterine cervical cancer patients with high-risk prognostic factors

**DOI:** 10.1007/s10147-018-01380-z

**Published:** 2018-12-22

**Authors:** Fumiaki Isohashi, Tadao Takano, Mamiko Onuki, Takahide Arimoto, Naoki Kawamura, Ryusuke Hara, Yoshiaki Kawano, Yukinobu Ota, Haruo Inokuchi, Hidenori Shinjo, Toshiaki Saito, Satoe Fujiwara, Takashi Sawasaki, Ken Ando, Koji Horie, Hiroyuki Okamoto, Naoya Murakami, Yoko Hasumi, Takahiro Kasamatsu, Takafumi Toita

**Affiliations:** 1 Department of Radiation Oncology, Osaka University Graduate School of Medicine, 2-2 (D10) Yamadaoka, Suita, Osaka 565-0871 Japan; 2 Department of Gynecology and Obstetrics, Tohoku University Graduate School of Medicine, Sendai, Japan; 3 Department of Obstetrics and Gynecology, Faculty of Medicine, University of Tsukuba, Tsukuba, Japan; 4 Department of Obstetrics and Gynecology, Graduate School of Medicine, The University of Tokyo, Tokyo, Japan; 5 Department of Gynecology, Osaka City General Hospital, Osaka, Japan; 6 Division of Radiation Oncology, Chiba Cancer Center, Chiba, Japan; 7 Department of Gynecology and Obstetrics, Graduate School of Medical Sciences, Kyushu University, Fukuoka, Japan; 8 Department of Gynecology, Osaka International Cancer Institute, Osaka, Japan; 9 Department of Radiation Oncology and Image-Applied Therapy, Graduate School of Medicine, Kyoto University, Kyoto, Japan; 10 Department of Radiation Oncology, Showa University Hospital, Tokyo, Japan; 11 Gynecology Service, National Kyushu Cancer Center, Fukuoka, Japan; 12 Department of Obstetrics and Gynecology, Osaka Medical College, Osaka, Japan; 13 Department of Gynecology, National Hospital Organization Kure Medical Center, Kure, Japan; 14 Department of Radiation Oncology, Gunma Prefectural Cancer Center, Gunma, Japan; 15 Department of Gynecology, Saitama Cancer Center, Saitama, Japan; 16 Department of Radiation Oncology, National Cancer Center Hospital, Tokyo, Japan; 17 Department of Obstetrics and Gynecology, Mitsui Memorial Hospital, Tokyo, Japan; 18 Department of Obstetrics and Gynecology, Tokyo Metropolitan Bokutoh Hospital, Tokyo, Japan; 19 Department of Radiology, Graduate School of Medical Science, University of the Ryukyus, Okinawa, Japan

**Keywords:** Cervical cancer, Cisplatin, Concurrent chemoradiotherapy, Postoperative, Three-dimensional radiotherapy

## Abstract

**Background:**

The aim of this study was to evaluate the effects of treatment with both three-dimensional radiotherapy (3DRT) and weekly 40-mg/m^2^ cisplatin on postoperative uterine cervical cancer patients with high-risk prognostic factors.

**Methods:**

We conducted a retrospective multi-institutional chart review of postoperative uterine cervical cancer patients with high-risk prognostic factors who had been treated with both 3DRT and weekly 40-mg/m^2^ cisplatin from 2007 to 2012. Each participating hospital provided detailed information regarding patient characteristics, treatment outcomes, and treatment complications.

**Results:**

The eligible 96 patients were analyzed. The median follow-up period was 61 months. The 3-year relapse-free survival, overall survival (OS), and locoregional relapse-free survival (LRFS) rates were 76%, 90%, and 88%, respectively. In multivariate analysis, the histological finding of either adenocarcinoma or adenosquamous carcinoma was a significant risk factor for both OS and LRFS. The percentage of patients with grade ≥ 3 acute hematologic toxicity, acute lower gastrointestinal toxicity (GIT), and late lower GIT were 45%, 19%, and 17%, respectively.

**Conclusions:**

The outcomes of concurrent chemoradiotherapy (CCRT) using weekly 40-mg/m^2^ cisplatin are similar to those in the previous studies that used several chemotherapy regimens. However, postoperative CCRT using 3DRT had a high level of late GIT.

## Introduction

On the basis of the results of a prospective randomized clinical trial, concurrent chemoradiotherapy (CCRT) has become the standard adjuvant treatment for surgically treated patients with early stage cervical cancer showing high-risk prognostic factors [positive pelvic lymph nodes (LNs), parametrial involvement, and/or positive surgical margin-commonly referred to as the “Peter’s criteria”] [1]. In this trial, CCRT using both cisplatin and 5-fluorouracil (5-FU) significantly improved both progression-free survival and overall survival (OS) compared with that of radiotherapy (RT) alone [1]. However, weekly 40-mg/m^2^ cisplatin is now considered a standard regimen. Cisplatin is used as a control arm in several clinical trials when used concurrently with RT [2–4], based on another trial [Gynecologic Oncology Group (GOG) 120] that found greater toxicity using concurrent cisplatin and 5-FU compared with that of cisplatin alone with definitive RT [5]. Patients undergoing conventional three-dimensional RT (3DRT) after radical hysterectomy experience considerable acute and chronic complications, including gastrointestinal toxicity (GIT), genitourinary toxicity (GUT), and hematologic toxicity (HT), because most tissues within the pelvic lesion are irradiated to the prescribed dose. Therefore, the use of intensity-modulated RT (IMRT) might be an attractive approach for reducing such toxicities. Several reports have indicated that IMRT can reduce radiation doses to the bladder, bowel, and bone marrow, and that IMRT is associated with lower rates of GUT, GIT, and HT compared with that of 3DRT [6–10]. However, each of these studies had limitations. Some studies had a limited number of patients [6, 7], some included patients with heterogeneous prognostic factors (intermediate- or high-risk prognostic factors) [6–9], and some included patients with various types of cancers (cervix or corpus) [10]. In addition, the clinical trial result that was recently reported [4], in which the primary endpoint was acute GIT, solved only one aspect of the problems, because the goal of using IMRT instead of 3DRT is not only to decrease the toxicity but also not to decrease the treatment outcomes. We conducted a prospective trial that evaluated CCRT using IMRT in patients who were surgically treated for early stage cervical cancer and showed high-risk prognostic factors (JCOG1402). These patients were recruited from the Japanese Clinical Oncology Group (JCOG), which is a nationwide study by Japanese oncologists. Unfortunately, the lack of appropriate prospective randomized trials and the small sample size of the published series have produced insufficient evidence for the historical control group of patients undergoing 3DRT. Therefore, we performed a retrospective analysis that evaluated 3DRT with weekly cisplatin in terms of both outcomes and complications in the participating groups, including the Gynecologic Cancer Study Group (GCSG) and Radiation Therapy Study Group (RTSG) of the JCOG to obtain highly detailed data on the historical control group before we initiated an IMRT trial.

## Patients and methods

### Study scheme

This retrospective study was based on a survey conducted by the investigators of JCOG1402. We conducted a retrospective multi-institutional chart review of postoperative uterine cervical cancer patients showing high-risk prognostic factors who were treated with both 3DRT and weekly 40-mg/m^2^ cisplatin in JCOG hospitals. IRB approval was obtained at each participating hospital. Each hospital provided detailed information regarding the patients’ characteristics, treatment outcomes, and treatment complications. Moreover, 34 centers in both the JCOG–GCSG and JCOG–RTSG agreed to participate in this study.

### Eligibility criteria

Postoperative uterine cervical cancer patients showing high-risk prognostic factors who were treated with both 3DRT and weekly 40-mg/m^2^ weekly cisplatin from January 2007 to December 2012 were enrolled in this study. Patients were eligible for the study if they met the following criteria: (1) clinical stage IB1–IIB cervical cancer (International Federation of Gynecology and Obstetrics 2008); (2) histologically confirmed positive pelvic LNs and/or parametrial invasion; (3) no distant metastasis before surgery; (4) no positive pathological para-aortic LNs; (5) had a radical hysterectomy; (6) no visible tumor or positive surgical margin; (7) histological findings of squamous cell carcinoma (SCC), adenocarcinoma (AD), or adenosquamous carcinoma (ADS); (8) aged between 20 and 70 years; (9) Eastern Cooperative Oncology Group score of 0–1; and (10) more than 20 dissected pelvic LNs.

### Radiotherapy

The clinical target volume (CTV) comprised a central vaginal CTV and a regional nodal CTV. The former included the proximal vagina and paravaginal tissues, and the latter included the common iliac, external and internal iliac, and presacral lymph nodes. An extended field RT was allowed for the patients with a positive high common iliac LNs. RT was given to a standard four-field box. The radiation source for treatment was 6MV or more. Additional brachytherapy was allowed.

### Statistical analysis

The relapse-free survival (RFS), OS, and locoregional relapse-free survival (LRFS) rates were calculated from the first day of CCRT to the day of any event or, if no event occurred, then to the day of last follow-up. The respective rates were estimated using the Kaplan–Meier method, and the differences were examined by the log-rank test. A Cox proportional hazard model was used for multivariate analysis. The relationship between clinical parameters and the incidence of complications was analyzed with the Fisher’s exact test. A receiver operating characteristic curve analysis was performed to select the most relevant threshold. A *p* value of < 0.05 was considered statistically significant. All statistical analyses were performed with R ver. 3.2.0 (The R Foundation for Statistical Computing, Vienna, Austria).

## Results

Data involving 119 patients were collected from 15 institutions of the JCOG group. The remaining 19 institutions had no eligible patients, because they used other adjuvant therapies for patients with high-risk prognostic factors. We excluded 23 ineligible patients, including 11 patients who did not show high-risk prognostic factors, 10 who had less than 20 dissected pelvic LNs, 1 who underwent IMRT, and 1 who was lost to follow-up. Finally, 96 patients were enrolled in the study for analysis. The median follow-up period was 61 months (range 8–107 months). Only 3 (3%) surviving patients had follow-up periods of ˂ 3 years. Patients and tumor characteristics are shown in Table 1. The median age of the patients was 43 years (range 27–69 years). Pathological findings indicated positive pelvic LNs in 84 patients (88%). The median number of positive LNs was 2 (range 1–58).

**Table 1:** Patient and tumor characteristics (*n* = 96)

	Median	SD
Age (years)	43	12
BMI (kg/m^2^)	21.0	3.1

	*n*	%
Smoker		
Yes	30	31
No/unknown	66	69
Diabetes		
Yes	2	2
No	94	98
FIGO stage		
IB1	43	45
IB2	21	22
IIA1/IIA2	5	5
IIB	27	28
Histology		
SCC	78	81
AD	12	13
ADS	6	6
Pelvic LNs		
0	12	13
1	26	27
2	25	26
≥ 3	33	34
Parametrial invasion		
Yes	49	51
No	47	49

*SD* standard deviation, *BMI* body mass index, *FIGO* International Federation of Gynecology and Obstetrics, *SCC* squamous cell carcinoma, *AD* adenocarcinoma, *ADS* adenosquamous carcinoma, *LN* lymph node

The treatment characteristics are shown in Table 2. The median number of LNs dissected at surgery was 42 (range 20–103). All patients received RT with ≥ 40 Gy, and 88% (84/96) of the patients received more than 4 cycles of chemotherapy.

**Table 2:** Treatment (*n* = 96)

	Median	Range
Dissected LNs	42	20–103
EBRT (Gy)	50	40–67
Interval between surgery and RT (days)	33	15–68
Course of chemotherapy	5	2–7

	*n*	%
Dose per fraction		
2 Gy	62	65
1.8 Gy	34	35
RT fields		
Whole pelvis	88	92
EFRT	8	8
ICBT		
Yes	14	15
No	82	85

*LN* lymph node, *EBRT* external beam radiotherapy, *RT* radiotherapy, *Gy* gray, *EFRT* extended field radiotherapy, *ICRT* intracavitary radiotherapy

**Fig. 1:**
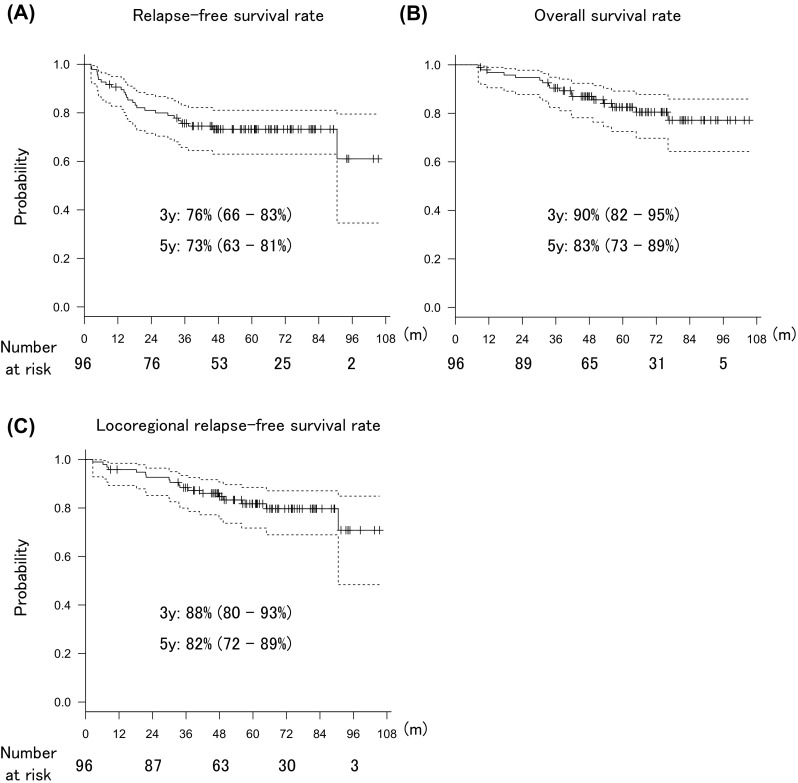
Kaplan–Meier analysis of relapse-free survival (**a**), overall survival (**b**), and locoregional relapse-free survival (**c**) in postoperative uterine cervical cancer patients with high-risk prognostic factors who were treated with both three-dimensional radiotherapy (3DRT) and weekly 40-mg/m^2^ cisplatin

The 3-year RFS, OS, and LRFS rates were 76% (95% CI 66–83%), 90% (95% CI 82–95%), and 88% (95% CI 80–93%), respectively, and the respective 5-year rates were 73% (95% CI 63–81%), 83% (95% CI 73–89%), and 82% (95% CI 72–89%) (Fig. 1). The treatment failure patterns were as follows: locoregional failure only in 4 patients, distant failure only in 18 patients, and both locoregional and distant failures in 4 patients. One patient experienced vaginal recurrence 5 years after surgery. Seven out of eight the patients had locoregional failure in the vagina or parametrium lesions.

Univariate analyses of the prognostic factors were performed with regard to RFS, OS, and LRFS (Table 3). The number of positive pelvic LNs (0–1 vs. ≥ 2) was a significant prognostic factor for RFS. Histology (SCC vs. AD/ADS) was a significant factor for both OS and LRFS. A multivariate analysis was performed with regard to histology, number of positive pelvic LNs, and parametrial invasion. In the multivariate analysis, histology was a significant risk factor for both OS and LRFS. The number of positive pelvic LNs tended to be a significant risk factor for RFS (*p* = 0.058) (Table 4).

**Table 3:** Univariate analysis of prognostic factors for RFS, OS, and LRFS in cervical cancer patients treated with post-surgical CCRT

	RFS				OS			LRFS		
	*n*	3-year RFS (%)	95% CI (%)	*p*	3-year OS (%)	95% CI (%)	*p*	3-year LRFS (%)	95% CI (%)	*p*
Age (years)										
< 42	45	71	55–82	0.535	86	72–94	0.814	82	67–90	0.863
≥ 42	51	80	66–89		94	83–98		94	83–98	
Smoker										
No	66	80	68–88	0.158	92	83–97	0.811	91	81–96	0.573
Yes	30	66	46–80		86	67–95		83	64–93	
FIGO										
IB–IIA	69	76	64–85	0.744	91	81–96	0.194	90	80–95	0.217
IIB	27	74	53–87		89	69–96		85	65–94	
Course of chemotherapy										
< 5	28	89	70–96	0.180	96	77–100	0.101	96	77–100	0.173
≥ 5	68	70	58–80		88	78–94		85	74–92	
Parametrial invasion										
No	47	78	64–88	0.396	94	81–98	0.345	89	76–95	0.317
Yes	49	73	58–83		87	74–94		88	74–94	
Pelvic LNs										
0–1	38	87	71–94	0.049	89	74–96	0.508	87	71–94	0.700
≥ 2	58	69	55–79		91	80–96		90	78–95	
Histology										
SCC	78	81	70–88	0.099	92	83–96	0.002	92	83–96	0.002
AD/ADS	18	55	29–74		83	57–94		72	46–87	
Dissected LNs										
< 40	43	72	55–83	0.498	86	71–93	0.533	81	66–90	0.322
≥ 40	53	79	65–88		94	83–98		94	83–98	

*RFS* relapse-free survival, *OS* overall survival, *LRFS* locoregional relapse-free survival, *CCRT* concurrent chemoradiotherapy, *FIGO* International Federation of Gynecology and Obstetrics, *LN* lymph node, *AD* adenocarcinoma, *ADS* adenosquamous carcinoma

**Table 4:** Multivariate analysis of prognostic factors for RFS, OS, and LRFS in patients with cervical cancer treated with postoperative CCRT

	RFS			OS			LRFS		
	HR	95% CI	*p*	HR	95% CI	*p*	HR	95% CI	*p*
Histology (SCC vs. AD/ADS)	1.9	0.8–4.5	0.119	4.3	1.6–11.4	0.003	4.2	1.6–10.8	0.002
Pelvic LN (0–1 vs. ≥2)	2.4	1.0–6.1	0.058	1.4	0.5–3.9	0.559	1.3	0.5–3.4	0.650
Parametrial invasion (no vs. yes)	1.5	0.7–3.3	0.294	1.9	0.7–5.2	0.192	1.8	0.7–4.7	0.216

*RFS* relapse-free survival, *OS* overall survival, *LRFS* locoregional relapse-free survival, *HR* hazard ratio, *CI* confidence interval, *SCC* squamous cell carcinoma, *AD* adenocarcinoma, *ADS* adenosquamous carcinoma, *LN* lymph node

Treatment-related toxicity was evaluated using the Common Terminology Criteria for Adverse Events Ver. 4.0. Table 5 presents the number of patients and grades of toxicity for HT, lower GIT, GUT, and lower extremity edema. The percentages of patients with grade ≥ 3 acute HT and lower GIT were 45% and 19%, respectively. The percentage of patients with grade ≥ 2 and grade ≥ 3 late lower GIT was 21% and 17%, respectively. All grade ≥ 2 late lower GIT occurred in the small bowel.

**Table 5:** Acute and late complications (*n* = 96)

Grade	2	3	4	≥ 3 (%)
Acute				
HT	–	38	5	45
Lower GIT	30	17	1	19
GUT	–	2	0	2
Others	–	3	0	3
Late				
Lower GIT	4	9	7	17
GUT	–	2	0	2
Lymphoedema	–	2	0	2
Others	–	2	1	3

*HT* hematologic toxicity, *GIT* gastrointestinal toxicity, *GUT* genitourinary toxicity

The incidence of grade ≥ 2 late GIT was analyzed as a function of clinical factors. The results of the univariate analysis are shown in Table 6. Age of ≥ 53 years was significantly associated with GIT.

**Table 6:** Univariate analysis of development of grade ≥ 2 late

	GIT		
	G0–1	G ≥ 2	*p*
BMI (kg/m^2^)			
< 21	36	12	0.315
≥ 21	40	8	
Smoker			
No	50	16	0.222
Yes	26	4	
Age (years)			
< 53	58	7	0.001
≥ 53	18	13	
RT field			
Whole pelvis	69	19	0.544
EFRT	7	1	
ICRT			
No	63	19	0.172
Yes	13	1	
Dissected LNs			
< 40	32	11	0.302
≥ 40	44	9	

*GIT* gastrointestinal toxicity, *BMI* body mass index, *RT* radiotherapy, *ICRT* intracavitary radiotherapy, *LN* lymph node

## Discussion

In this study, we retrospectively evaluated postoperative uterine cervical cancer patients showing high-risk prognostic factors who were treated with both 3DRT and weekly 40 mg/m^2^ cisplatin. There are few reports in the literature on such patients, although weekly 40 mg/m^2^ cisplatin has been considered a standard regimen when used concurrently with RT. To our knowledge, this is the largest study on postoperative uterine cervical cancer patients showing high-risk prognostic factors who were treated with both 3DRT and weekly 40 mg/m^2^ cisplatin. The 3-year and 5-year OS rates were 90% and 83%, respectively, and the 3-year and 5-year RFS rates were 76% and 73%, respectively. The outcomes in the current study are similar to those in the previous studies that used several multiagent chemotherapy regimens and showed 3-year and 5-year OS rates of 84–87% and 79–81%, or 3-year and 5-year RFS rates of 76–84% and 70–77%, respectively [1, 11–13]. Thus, this study reconfirmed that CCRT using weekly 40 mg/m^2^ cisplatin is the standard treatment regimen.

The percentage of patients with grade ≥ 3 late lower GIT was 17%. The incidence rate of late GIT in the current study is similar to or even higher than that in the previous CCRT studies that used several chemotherapy regimens and showed the incidence rate of grade ≥ 3 late GI to be 6–19% [1, 3, 6, 7, 11–14]. One of the reasons for the higher late GIT may be the method of radical hysterectomy in Japan. Japanese patients are generally slimmer in build than that of patients in the West, as evidenced by the median BMI in the current study, which was 21.0. Therefore, it may be assumed that a hysterectomy is more extensive, and lymphadenectomy is more systemic in Japan, as evidenced by the median number of dissected LNs in the current study, which was 42. The number of dissected LNs was higher than that of the overseas reports [15, 16]. Consequently, Japanese gynecologic oncologists have a tendency to avoid adjuvant CCRT, because they are worried that patients undergoing CCRT may experience severe GIT. In fact, 15% (5/34) of the institutions selected chemotherapy alone and 6% (2/34) of the institutions selected RT alone as the adjuvant therapy. A survey from institutions belonging to the Japanese Gynecology Oncology Group (JGOG) showed that 72% of the institutions selected chemotherapy alone for postoperative cervical cancer patients having intermediate/high-risk factors [17]. Several phase II studies in Japan have indicated that chemotherapy alone had a comparable survival outcome and lower toxicity compared to CCRT for postoperative cervical cancer having high-risk factors [18, 19]. Therefore, the JGOG is going to launch in the future a phase III study comparing postoperative CCRT to chemotherapy alone in surgically treated high-risk stage IB–IIB cervical cancer patients. In addition, Trifietti et al. reported that, in a large cohort of women with high-risk cervical cancer in the US, ˂50% women received adjuvant CCRT, and the use of adjuvant CCRT did not significantly increase between 2002 and 2012 [20]. This may have happened, because the gynecologic oncologists in the US are also worried about radiation complications. Therefore, the evaluation of adjuvant IMRT, which has a potential for decreasing complications without reducing outcomes, for cervical cancer with high-risk prognostic factors, is very important. As stated above, we conducted a multicenter prospective trial that evaluated CCRT using IMRT in such high-risk patients. There have been some reports, including ours, suggesting that the elderly patients have a higher incidence of GIT after postoperative RT [21, 22]. A logistic regression curve analysis indicated that the probability of late GIT was increased as the patients becoming older (*p* = 0.009) (data not shown). It appears that the intestine of older patients is more vulnerable to pelvic RT.

Univariate and multivariate analyses showed that AD/ADS histology and number of positive pelvic LNs (≥ 2) were predictive of worse outcomes.

These results indicated that CCRT using weekly 40 mg/m^2^ cisplatin for patients with these risk factors has poor treatment outcomes. A possible strategy that might improve the outcomes in these patients is to use stronger chemotherapy with RT, including the concurrent use of platinum-based doublet chemotherapy and/or the addition of consolidation chemotherapy after adjuvant CCRT. Lee et al. reported that the 5-year RFS and OS rates were 77% and 80%, respectively, in patients with surgically treated high-risk cervical cancer who received carboplatin plus paclitaxel (TC)-based CCRT using 3DRT [11]. Mabuchi et al. reported excellent outcomes in a prospective study on high-risk cervical cancer patients that were treated with combined TC with IMRT followed by TC-based consolidation chemotherapy [23]. Their results demonstrated that the 3-year progression-free survival and OS rates were 89% and 94%, respectively. In addition, a randomized trial (GOG 0724) is ongoing to evaluate the role of consolidation TC chemotherapy after weekly cisplatin-based CCRT in patients with surgically treated high-risk cervical cancer. These results could help to identify patients who would benefit from these high-intensity regimens in the future. In addition, in this study, seven out of eight patients had locoregional failure in the vagina or parametrium lesions. Therefore, this suggests that the need of dose escalation in the vagina or parametrium lesions, and simultaneous integrated boost IMRT might be an attractive approach for increasing the dose to the vagina or parametrium lesions.

In conclusion, the outcomes of CCRT using weekly 40 mg/m^2^ cisplatin are similar to those in the previous studies that used several chemotherapy regimens. However, CCRT using 3DRT had a high level of late GIT, and further investigation is needed to evaluate the safety and efficacy of pelvic RT using the IMRT technique instead of 3DRT.
